# Macular hole and vitreous hemorrhage subsequent to stereotactic hypofractionated radiotherapy for choroidal melanoma: A case report and review of the literature

**DOI:** 10.3389/fonc.2022.1060307

**Published:** 2022-11-22

**Authors:** Xiaoyin Zhou, Hiroto Ishikawa, Fumi Gomi

**Affiliations:** ^1^ Department of Ophthalmology, Hyogo College of Medicine, Hyogo, Japan; ^2^ Department of Ophthalmology, Mirai Eye & Skin Clinic, Osaka, Japan

**Keywords:** choroidal melanoma, stereotactic hypofractionated radiotherapy, macular hole, vitreous hemorrhage, case report, pars plana vitrectomy

## Abstract

Choroidal melanoma is the leading primary intraocular tumor with potentially fatal outcomes in adults. The coexistence of choroidal melanoma and a macular hole is extremely rare, and treatment strategies and information on the prognosis of associated complications are currently lacking. We report the first case of choroidal melanoma complicated with a macular hole and vitreous hemorrhage after stereotactic hypofractionated radiotherapy in Japan, and review the relevant literature in relation to the possible mechanisms, treatment strategies, and outcomes. An 83-year-old male with choroidal melanoma was treated with stereotactic hypofractionated radiotherapy in January 2021. Five months later, a full-thickness macular hole developed, followed by an acute massive vitreous hemorrhage about 2 weeks later. Following confirmation of tumor regression, the patient underwent a pars plana vitrectomy and internal limiting membrane peeling. The macular hole was closed postoperatively and the patient’s best-corrected visual acuity improved to 20/125. There was no evidence of intraocular tumor dissemination or distant metastases during follow-up. A systematic literature search only identified 10 previous cases of choroidal melanoma with a macular hole in eight reports worldwide, mainly in females. Macular edema may be the primary cause of macular hole formation in these cases. Most patients who underwent vitrectomy for complications after tumor regression achieved a good prognosis. The development of a macular hole is a rare complication associated with choroidal melanoma. Anterior-posterior traction of posterior vitreous detachment and secondary macular edema may have contributed to the formation of the macular hole in the current case.

## Introduction

Choroidal melanoma is the leading primary intraocular malignancy among adults (1), with a low incidence of 0.6 cases per million per year in Japan (2). However, considering the high mortality rate of malignant metastases, this life-threatening disease should be diagnosed and treated promptly. Radiation therapy, including plaque brachytherapy, proton beam radiotherapy, and stereotactic radiotherapy, is an alternative to enucleation and has become the first-line treatment for choroidal melanoma (3–5). However, the tumor may be accompanied by complications, such as vitreous hemorrhage, rhegmatogenous retinal detachment, and macular hole (MH). Care is therefore needed to prevent intraocular or extraocular tumor dissemination during therapy for these complications (6).

Choroidal melanoma coexisting with a MH is extremely rare. To the best of our knowledge, only 10 previous cases have been reported worldwide (6–13), none of which occurred after stereotactic hypofractionated radiotherapy, and with limited information on the treatment of associated complications. Herein, we report on a patient who was diagnosed with asymptomatic choroidal melanoma with atypical presentation, and who developed a full-thickness MH and vitreous hemorrhage during follow-up, which was eventually repaired by pars plana vitrectomy (PPV) with internal limiting membrane (ILM) peeling. We also reviewed the relevant literature regarding the possible mechanisms of MH formation in patients with choroidal melanoma, and the corresponding treatment management and outcomes.

## Case presentation

**Figure 1:**
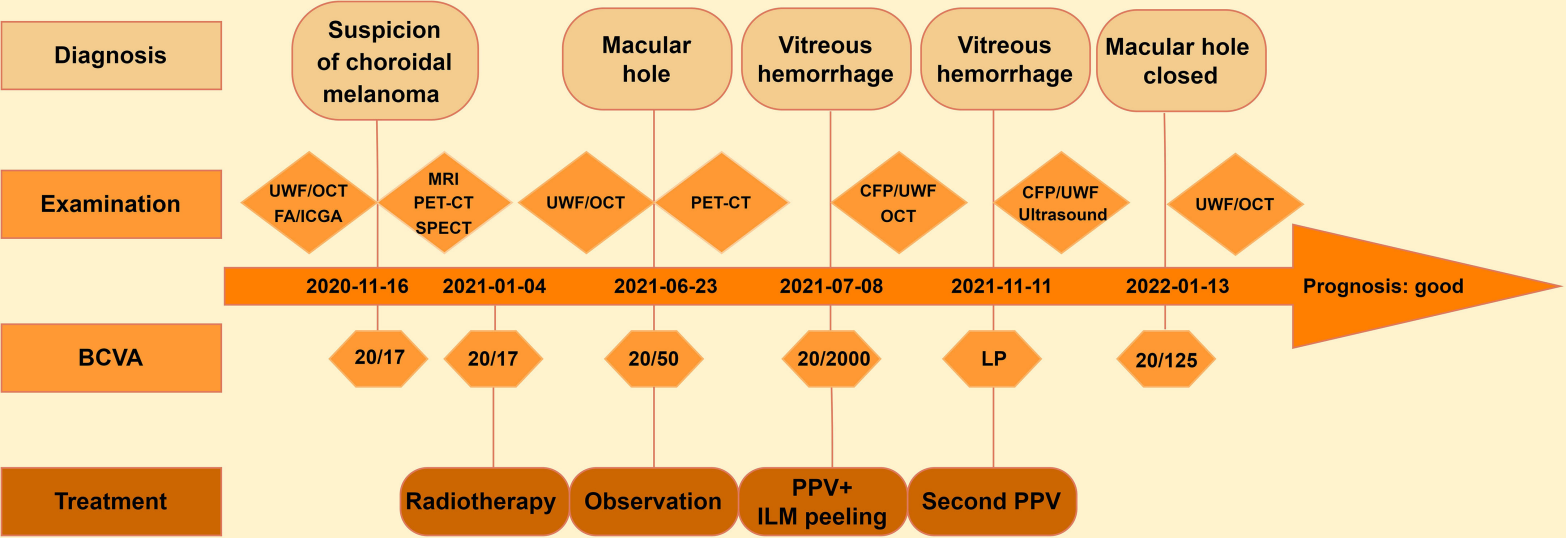
Clinical course of the patient with choroidal melanoma. UWF, ultra-wide-field fundus photography; OCT, optical coherence tomography; FA, fluorescein angiography; ICGA, indocyanine green angiography**;** MRI, magnetic resonance imaging; PET-CT, positron emission tomography-computed tomography; SPECT, single-photon emission computed tomography; CFP, color fundus photography; BCVA, best-corrected visual acuity; LP, light perception; PPV, pars plana vitrectomy; ILM, internal limiting membrane.

**Figure 2:**
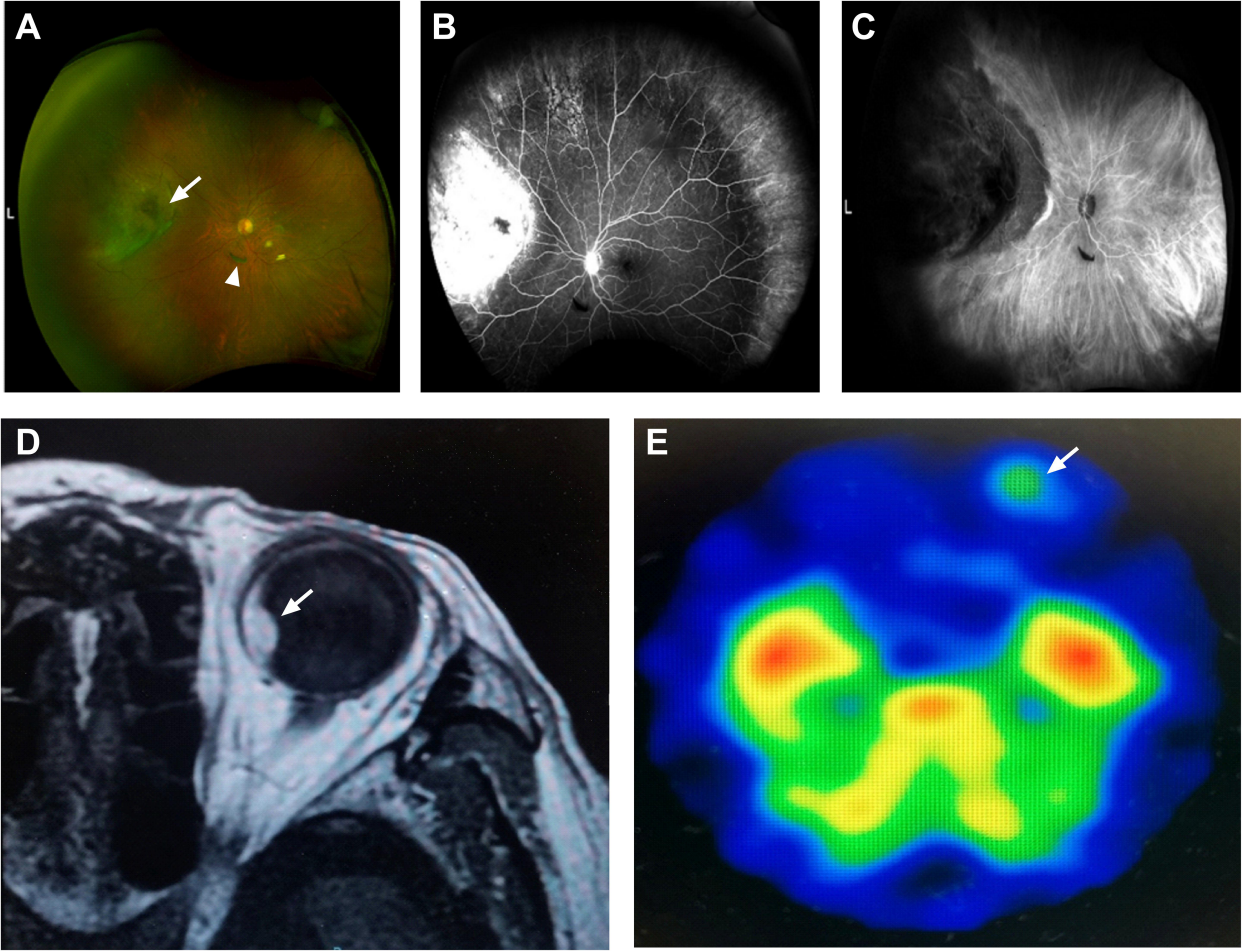
An 83-year-old man with choroidal melanoma. **(A)** Ultra-wide-field fundus photography revealed a white, elevated choroidal tumor with a central dark brown speckle (arrow) in the nasal quadrant and posterior vitreous detachment (arrowhead) inferior to the optic nerve. **(B)** Fluorescein angiography showed early hyperfluorescence corresponding to the mass. **(C)** Indocyanine green angiography showed blocked fluorescence and a small hyperfluorescent area at the edge. **(D)** Magnetic resonance imaging demonstrated a hyperintense tumor (arrow) with a smooth border on axial T1 imaging-fast spin-echo. **(E)** Iodine-123 isopropyl iodoamphetamine brain single-photon emission computed tomography showed high uptake in his left eye (arrow).

An 83-year-old man was referred to our hospital with suspected serous retinal detachment in his left eye. The patient’s clinical course is presented in **Figure 1**. The best-corrected visual acuity (BCVA) was 20/20 in his right eye and 20/17 in his affected left eye. The intraocular pressure was normal (14 mmHg in the right eye and 13 mmHg in the left eye), and there were no appreciable findings in the anterior segments. Ultra-wide-field fundus photography (**Figure 2A**) of the left eye revealed an elevated choroidal mass with a central dark brown speckle in the nasal quadrant, about 4 disc diameters from the optic disc, along with concomitant posterior vitreous detachment (PVD). Fluorescein angiography showed that the choroidal mass had early diffuse hyperfluorescence with a central area of hypofluorescence (**Figure 2B**). Indocyanine green angiography showed blocked fluorescence due to the choroidal mass and a small hyperfluorescent area at the margin in the late stage (**Figure 2C**). Magnetic resonance imaging demonstrated a tumor measuring 5.9×5.7 mm in basal dimensions and 4.1 mm thick, with a hyperintense signal toward the vitreous cavity on axial T1 imaging-fast spin-echo (**Figure 2D**). Iodine-123 isopropyl iodoamphetamine brain single-photon emission computed tomography revealed high focal uptake in his left eye, corresponding to the choroidal tumor (**Figure 2E**). The patient underwent integrated positron emission tomography/computed tomography, and a transaxial section across the left eye revealed no fluorodeoxyglucose activity and no evidence of distant metastases. There were no abnormalities in the fellow eye.

**Figure 3:**
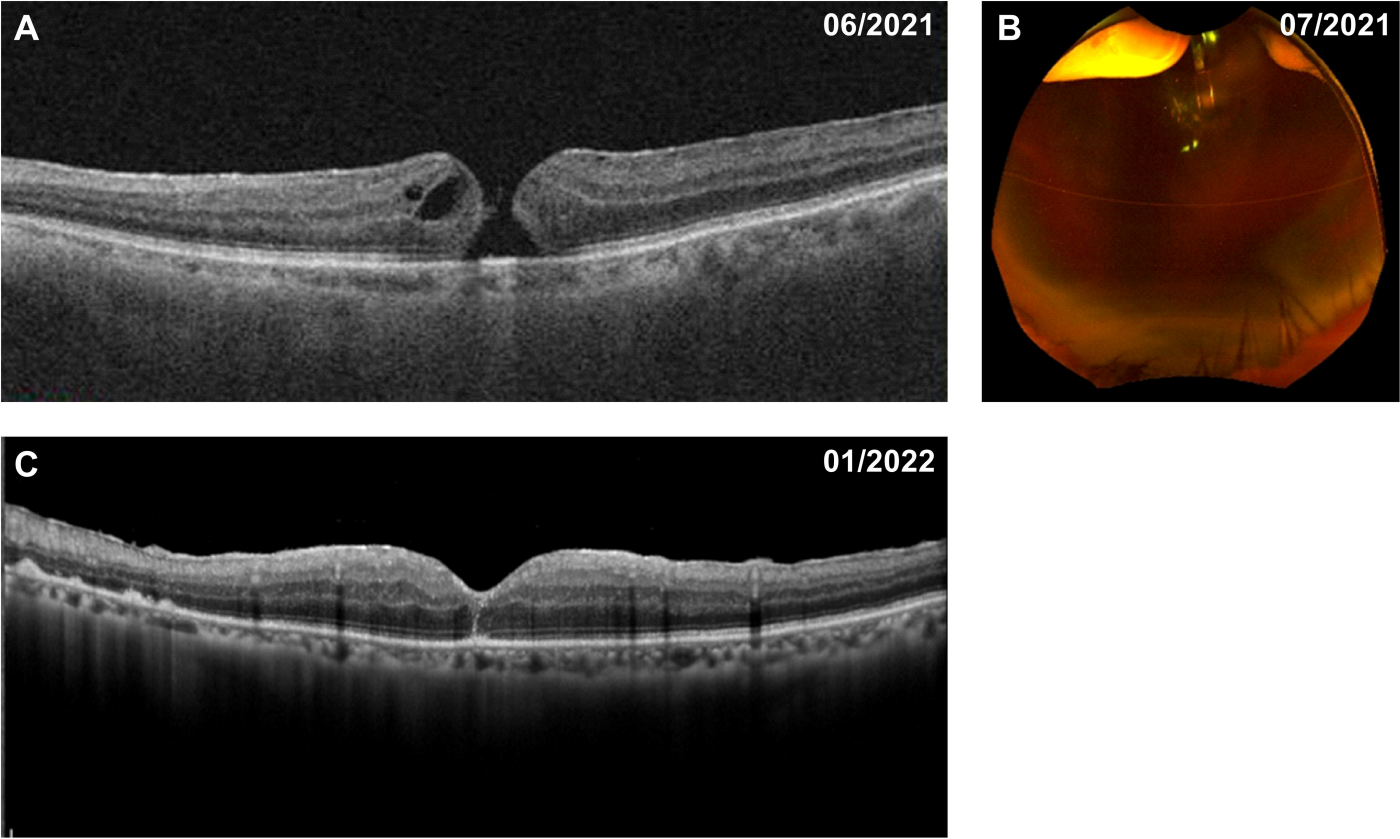
Fundus appearance in the patient with choroidal melanoma before and after vitrectomy. **(A)** Five months after radiotherapy, a full-thickness macular hole with cystic cavities was shown on optical coherence tomography. **(B)** A further 2 weeks later, a massive vitreous hemorrhage appeared on ultra-wide-field fundus photography. **(C)** The macular hole was successfully closed after pars plana vitrectomy with internal limiting membrane peeling.

The patient received a course of stereotactic hypofractionated radiotherapy (60 Gy in 5 fractions) for 5 consecutive days after the clinical diagnosis of choroidal melanoma. Five months later, the patient complained of visual deterioration with a BCVA of 20/50 and distortion in his left eye. Fundus examination and optical coherence tomography showed a full-thickness MH (stage 4) with cystic cavities (**Figure 3A**). Approximately 2 weeks later, his BCVA had decreased to 20/2000, attributed to an acute massive vitreous hemorrhage (**Figure 3B**). Repeat positron emission tomography/computed tomography examination showed no significant abnormalities or metastases. We therefore performed a vitrectomy and inverted ILM peeling. During surgery, we found a massive subretinal hemorrhage, abundant fibrin, and retinal fragility but no obvious tears in his left eye. Gas-fluid exchange was completed at the end of surgery using 20% sulfur hexafluoride. After the first vitrectomy, the MH was closed on optical coherence tomography examination. However, the vitreous hemorrhage reappeared 2 weeks later and we performed a second vitrectomy after 4 months of observation. The MH remained closed after the two procedures (**Figure 3C**), the retina remained attached, the tumor displayed marked regression, and the BCVA had improved to 20/125.

## Review of the literature

We conducted a literature review by searching the PubMed, Cochrane Library, and Web of Science databases using the keywords (“choroidal melanoma” OR “uveal melanoma”) AND (“macular hole” OR “retinal tear”), (“uveal neoplasms” OR “ choroidal neoplasms”) AND (“macular hole” OR “retinal tear”), for articles published from December 1951 to March 2022. The search was limited to publications in English. We reviewed the abstracts and full texts of the identified articles and the related references. Simultaneous occurrence of choroidal melanoma and MH was reported in 10 patients in eight publications (6–13), after excluding one case with MH in which it was difficult to determine the intraocular tumor type (14) and one case in which MH was considered a post-vitrectomy complication (15). The findings of the literature review and the current case are presented in **Table 1**.

**Table 1:** Review of previously reported cases of choroidal melanoma with macular hole and the current case.

	Country	Age/Sex/Eye	Initial visual acuity	Tumor location to the optic disc	Tumor base (mm)	Tumor thickness (mm)	Tumor color	Tumor treatment	Time of macular hole	Macular hole diameter (µm)	Other manifestations	Procedure	Macular hole/Metastases
Zhou XY	Japan	83/M/OS	20/17	Nasal	5.9 × 5.7	4.1	Amelanotic	Stereotactic hypofractionated radiotherapy	5M after radiotherapy	232	PVD; VH	PPV+ILMP	Closed/No
Foster WJ (6)	America	84/M/OS	NA	Anterior to the equator	7 × 6	3.5	NA	Plaque	After Plaque	NA	NA	PPV+ILMP	NA/No
Gold AS (7)	America	65/F/OS	20/400	Temporal	16 × 14	2.4	Melanotic	Plaque	Concurrence	NA	RD	PPV+ILMP	Closed/No
Gold AS (7)	America	77/F/OS	20/200	Nasal	14 × 13	2.3	Melanotic	Plaque	Concurrence	NA	CME	No	Unclosed/No
Gold AS (7)	America	70/F/OD	20/200	Inferonasal	16 × 14	5.8	Melanotic	Plaque	NA	NA	CME	No	NA/Yes
Narang S (8)	India	45/F/OS	20/1200	Temporal	10.2 × 9.1	4.7	Orangeish-yellow	TTT	Concurrence	500	RD; CME	No	NA/No
Balestrazzi A (9)	Italy	62/F/OS	20/20	Superior	8.2 × 7.7	2.5	Pigmented	TTT	3M after TTT	NA	RD	PPV+ILMP	Closed/No
Uffer S (10)	Switzerland	71/M/OS	HM	Temporal	NA	13.0	Pigmented	Enucleation	Concurrence	NA	RD; CME; PVD	No	NA/No
Shields CL (11)	America	75/F/OD	20/100	Superonasal	NA	NA	Melanotic	Plaque	26M after Plaque	NA	DH; CME	STTA	Unclosed/NA
Beykin G (12)	Israel	72/F/OS	FC 30cm	NA	6.4 × 7.2	2.6	NA	Plaque	After Plaque	NA	RD	PPV+ILMP	Closed/No
Damato B (13)	England	45/M/OS	NA	Nasal	8 × NA	8	NA	Local resection	After resection	NA	RD	PPV	Unclosed/NA

OD, right eye; OS, left eye; HM, hand motion; FC, finger counting; TTT, transpupillary thermotherapy; Plaque: iodine-125 plaque brachytherapy; PVD, posterior vitreous detachment; VH, vitreous hemorrhage; RD, retinal detachment; CME, cystoid macular edema; DH, disk hemorrhage; PPV, pars plana vitrectomy; ILMP, internal limiting membrane peeling; STTA, subtenon triamcinolone acetonide injection; NA, not available.

The total of 11 patients included four males and seven females, with an average age of 68.1 ± 13.2 years (range, 45–84 years). Five cases were reported in America, three in Europe (England, Italy, Switzerland), and two in West Asia (India, Israel). The current case was first documented in Japan (East Asia). The mean tumor-base diameter was 10.2×9.6 mm and the mean thickness was 4.9 mm. Most cases had a melanotic appearance, but the present case appeared amelanotic. Regarding the treatment of the tumor, one case underwent enucleation due to the large size of the tumor (height 13 mm) and total retinal detachment (10), one case underwent transscleral local resection (13), and the remaining cases were treated with radiotherapy, including stereotactic hypofractionated radiotherapy (the only treatment in the current case). MH developed in five cases after radiotherapy and in one case after resection, and was observed in four cases at melanoma diagnosis. The most common concomitant manifestations during the follow-up period were retinal detachment and macular edema; the present case was the only one in which PVD and massive vitreous hemorrhage were observed. Five cases with MH were repaired with PPV and ILM peeling and achieved good results, except for the absence of detailed information on the macular prognosis in one case (6) and one case that was unsuccessfully repaired by PPV and eventually underwent enucleation (13). Two cases that underwent observation (7) and subtenon triamcinolone acetonide injection (11), respectively, ended in failure of macular closure. One case reported by Gold et al. died of suspected metastatic disease (7), but no recurrence or metastases were reported in the remaining cases.

## Discussion

Uveal melanoma is a severe intraocular malignancy with an elevated gray or gray-brown appearance, which predominantly occurs in Caucasians and is generally complicated with exudative retinal detachment and occasional vitreous hemorrhage (1, 16). Rarely, MH can develop, and appropriate procedures must be followed to avoid metastasis (7). We presented a case with an atypical manifestation of choroidal melanoma with MH and subsequent vitreous hemorrhage in a patient who achieved a good prognosis after treatment.

Full-thickness MH represents an anatomical defect in the fovea involving interruption of all the retinal layers from the ILM to the retinal pigment epithelium (17). Notably, MH is more prevalent in women over the age of 60 years, due to hormonal influences (18), which may explain the high proportion of women in the above case series. There are several hypotheses regarding the co-occurrence of melanoma and MH. Typically, the development of MH in the retina has been attributed to anterior-posterior traction, commonly induced by PVD (19). In our case, PVD was detected by fundus observation at the first diagnosis, with no obvious retinal break. Although the specific etiology was unclear, we presumed that the PVD might have been caused by chronic tumor-related vitreous inflammation or floating blood cells, which could induce vitreous condensation, liquefaction, and final separation from the retina. In elderly patients, PVD may be caused by an inevitable, complex series of events such as synchysis and syneresis in the vitreous (17). It is therefore also possible that normal age-related PVD with secondary MH may have occurred coincidentally with melanoma in our case. In addition, tangential traction possibly resulting from an epiretinal membrane or increasing tumor height/thickness causing a lateral shift of vitreomacular traction may also play an important role in the pathogenesis of MH (20), as in a prior case report (7).

However, degenerative etiologies such as cystoid macular edema (CME) and secondary rupture of cysts may be responsible for the formation of MH, as seen in four previous reports (7, 8, 10, 11). Retinal degeneration overlying the tumor, intraretinal edema secondary to chronic exudative retinal detachment, or an inflammatory cellular reaction to the necrotic tumor in the vitreous may lead to the development of CME in patients with melanoma (21). In one case in which peripheral melanoma was associated with CME, trypsin digest preparation of the intraretinal space demonstrated abnormal capillary architecture, which was thought to account for vascular leakage (22). CME may have been caused by an increase in capillary permeability during inflammation, resulting in MH (8). Similarly, we detected cystic cavities in the current case, suggesting that tractional forces followed by retinal tissue degeneration at the macula may have facilitated the formation of the MH.

Stereotactic hypofractionated radiotherapy, iodine-125 plaque brachytherapy, and transpupillary thermotherapy (TTT) are possible options for globe-salvaging treatment in patients with peripherally located tumors (3, 23–25). However, ocular complications such as cataract, glaucoma, maculopathy, and optic neuropathy require prompt attention (26). Mashayekhi et al. reported that most cases of atrophic retinal holes in the TTT-treated area occurred within 6 months after treatment, while retinal atrophy was much less prominent in patients treated with plaque radiotherapy or stereotactic hypofractionated radiotherapy (27, 28). Heat-induced vitreous changes in TTT may lead to vitreoretinal traction or retinal atrophy, which may in turn explain the formation of a retinal hole (27). Balestrazzi et al. described a case of MH that occurred 3 months after TTT in a patient with melanoma. However, given the distance between the tumor and the macula, TTT was considered unlikely to have caused the MH in this case (9). In another case report, Beykin et al. observed an atrophic MH in close proximity to a melanoma after plaque radiotherapy (12). More than 50% of patients in one study cohort suffered late-onset radiation retinopathy 5 years after stereotactic hypofractionated radiotherapy (28). Another study found that the distance from the fovea to the tumor was the primary determinant of maculopathy in patients undergoing radiotherapy (26). In the current case, MH was observed 5 months after stereotactic hypofractionated radiotherapy, and the melanoma site was distant from the macula, thus ruling out the possibility of radiation-induced MH. However, long-term complications still require cautious evaluation in this case.

The incidences of vitreous hemorrhage in patients with uveal melanoma treated with plaque radiotherapy were 15.1% at 5 years and 18.6% at 10 years (29). Radiation can lead to fibrosis and necrosis of tumor tissue, as well as thinning and fragility of the retina, thus increasing the risk of bleeding. Radiation-induced tumor necrosis is most commonly linked to vitreous hemorrhage in melanoma-affected eyes after radiotherapy, followed by proliferative radiation retinopathy and PVD (29). Combined with the surgical finding of bleeding from the tumor surface, we considered that the hemorrhage in the present case was a consequence of acute ischemic shrinkage of the tumor after stereotactic hypofractionated radiotherapy and vascular rupture within the tumor. However, it is important to note that the occurrence of vitreous hemorrhage before melanoma treatment should raise concerns about possible tumor invasion through Bruch’s membrane and diffuse intraocular tumor dissemination (6).

Limited information is available on the treatment of MH and vitreous hemorrhage in eyes with choroidal melanoma. Hypofractionated stereotactic radiotherapy with 50–70 Gy in five fractions or plaque brachytherapy has recently proven sufficient to preserve the eyeball and achieve excellent local tumor control in patients with choroidal melanoma (28, 30). Among these 11 cases we reviewed, two cases did not explicitly state whether a metastasis occurred and one case was unclear whether the macular hole developed before or after radiation treatment, but was lost to follow-up and metastatic disease was presumed. In the remaining cases, no metastases were found during follow-up. Besides, almost all eyes with treated melanoma had a good prognosis in terms of the MH after PPV and ILM peeling treatment. Beykin et al. retrospectively evaluated six patients with radiation-treated choroidal melanoma who developed retinal detachment and one who developed MH, all of whom underwent PPV and ultimately had attached retinas (12). During a 5-year follow-up period, Bianciotto et al. revealed that the resolution rate of vitreous hemorrhage in regressed melanoma eyes was as high as 72% after vitrectomy, and PPV did not increase the risk of tumor recurrence or distant metastasis, with low rates of 3% and 5%, respectively (29). Exceptionally, Foster et al. reported one patient with vitreous hemorrhage before tumor treatment who unfortunately developed intraocular tumor spread after PPV, while the remaining eight patients with tumor regression developed complications including vitreous hemorrhage, MH, or retinal detachment, but showed no tumor spread following PPV (6). Therefore, we consider the complication that happened before tumor treatment will increase the risk of metastasis. In contrast, metastasis is comparatively low if a complication occurs following tumor remission. The timing of vitrectomy in our case was 6 months after tumor radiotherapy and the shortest interval was 3 months in a previous case; with no evidence of tumor dissemination in either case during follow-up (9). The conservative interval for vitrectomy after tumor treatment is unclear, but definite tumor regression should be confirmed before carrying out vitrectomy or other intraocular surgery. Furthermore, direct contact with the tumor or direct instrument interaction should be minimized and all steps should be carried out carefully during surgery.

The expectation of visual improvement also needs to be considered, especially in patients with chronic MH. Two cases of choroidal melanoma still had poor vision after MH repair surgery (7, 12). Conversely, another case who developed MH 3 months after TTT had improved visual acuity from hand motion to 20/80 after timely vitrectomy (9), similar to the current case. PPV after confirmed tumor regression thus seems to be a feasible and effective treatment in terms of anatomical and visual success, and may be more beneficial in cases with newly developed MH. A recent case report notably demonstrated extraocular extension of a brachytherapy-treated choroidal melanoma following PPV and scleral buckle for rhegmatogenous retinal detachment (31). Combined with previous reports, PPV, especially with ILM peeling, might increase the risk of tumor recurrence or migration of tumor cells, but the risks of these surgical complications in patients with regressed tumors are probably low (6). However, minimizing visual loss and preventing metastasis of malignant tumors still require careful assessment to balance the risks and benefits.

In conclusion, there have been few reports of choroidal melanoma complicated with MH and vitreous hemorrhage in the literature. Vitrectomy seems to be feasible for repairing MH in patients with regressed tumors. However, the occurrence of complications after intraocular tumor treatment and the safety of vitrectomy for these complications require longer follow-up and cautious management.

## Data availability statement

The raw data supporting the conclusions of this article will be made available by the authors, without undue reservation.

## Ethics statement

Ethical review and approval was not required for the study on human participants in accordance with the local legislation and institutional requirements. The patients/participants provided their written informed consent to participate in this study. Written informed consent was obtained from the individual(s) for the publication of any potentially identifiable images or data included in this article.

## Author contributions

HI conceived the idea for the article. XZ performed the literature review and drafted the manuscript. HI and FG revised and approved the final version of the manuscript.

## Funding

This study was supported by Hyogo Medical University Diversity Grant for Research Promotion” under MEXT Funds for the Development of Human Resources in Science and Technology, Initiative for Realizing Diversity in the Research Environment (Characteristic-Compatible Type).

## Conflict of interest

The authors declare that the research was conducted in the absence of any commercial or financial relationships that could be construed as a potential conflict of interest.

## Publisher’s note

All claims expressed in this article are solely those of the authors and do not necessarily represent those of their affiliated organizations, or those of the publisher, the editors and the reviewers. Any product that may be evaluated in this article, or claim that may be made by its manufacturer, is not guaranteed or endorsed by the publisher.

## References

[1] Jager MJ Shields CL Cebulla CM Abdel-Rahman MH Grossniklaus HE Stern MH . Uveal melanoma. Nat Rev Dis Primers (2020) 6:18–20. doi: 10.1038/s41572-020-0158-0 32273508

[2] Tomizuka T Namikawa K Higashi T . Characteristics of melanoma in Japan: A nationwide registry analysis 2011-2013. Melanoma Res (2017) 27:492–7. doi: 10.1097/CMR.0000000000000375 28609317

[3] Reichstein DA Brock AL . Radiation therapy for uveal melanoma: a review of treatment methods available in 2021. Curr Opin Ophthalmol (2021) 32:183–90. doi: 10.1097/ICU.0000000000000761 33770014

[4] Thornton S Coupland SE Heimann H Hussain R Groenewald C Kacperek A . Effects of plaque brachytherapy and proton beam radiotherapy on prognostic testing: a comparison of uveal melanoma genotyped by microsatellite analysis. Br J Ophthalmol (2020) 104:1462–6. doi: 10.1136/bjophthalmol-2019-315363 32024655

[5] Tsai YC Kuo CY Lin JW Yang ST Lai SC Tsai JT . Gamma knife perfexion® radiosurgery and endo diode laser thermotherapy for choroidal melanoma with technical analysis: a case report. Oncol Lett (2018) 15:91–8. doi: 10.3892/ol.2017.7300 PMC5766057 29375706

[6] Foster WJ Harbour JW Holekamp NM Shah GK Thomas MA . Pars plana vitrectomy in eyes containing a treated posterior uveal melanoma. Am J Ophthalmol (2003) 136:471–6. doi: 10.1016/S0002-9394(03)00244-7 12967800

[7] Gold AS Bermudez E Latiff A Wildner AC Ehlies FJ Murray TG . Posterior uveal melanoma coexistent with macular hole. Optom Vis Sci (2013) 90:156–60. doi: 10.1097/OPX.0b013e3182924a9b 23604299

[8] Narang S Kochar S Pannu KS Kalra N Gupta R Sood S . Choroidal melanoma with macular hole. Indian J Ophthalmol (2004) 52:238–41. Available at: https://www.ijo.in/text.asp?2004/52/3/238/14582 15510467

[9] Balestrazzi A Blasi MA Scupola TA Balestrazzi TE . Retinal detachment due to macular hole after transpupillary thermotherapy of choroidal melanoma. Retina (2001) 21:384–5. doi: 10.1097/00006982-200108000-00019 11508891

[10] Uffer S Zografos L . Macular hole in a case of choroidal melanoma. Eur J Ophthalmol (1997) 7:115–8. doi: 10.1177/112067219700700121 9101207

[11] Shields CL Demirci H Marr B Mashayekhi A Dai V Materin M . Intravitreal triamcinolone acetonide for acute radiation papillopathy. Retina (2006) 26:537–44. doi: 10.1097/00006982-200605000-00007 16770260

[12] Beykin G Pe’er J Hemo Y Frenkel S Chowers I . Pars plana vitrectomy to repair retinal detachment following brachytherapy for uveal melanoma. Br J Ophthalmol (2013) 97:1534–7. doi: 10.1136/bjophthalmol-2013-303331 24064939

[13] Damato B Groenewald CP McGalliard JN Wong D . Rhegmatogenous retinal detachment after transscleral local resection of choroidal melanoma. Ophthalmology (2002) 109:2137–43. doi: 10.1016/S0161-6420(02)01240-X 12414429

[14] Gündüz AK Mirzayev I Ceyhan K Özalp Ateş FS . Transretinal biopsy *via* 23-gauge pars plana vitrectomy for retinal and choroidal tumors: cytopathological results, surgical complications, and patient outcomes. Jpn J Ophthalmol (2021) 65:250–60. doi: 10.1007/s10384-020-00795-4 33420856

[15] McCannel TA McCannel CA . Iodine 125 brachytherapy with vitrectomy and silicone oil in the treatment of uveal melanoma: 1-to-1 matched case-control series. Int J Radiat Oncol Biol Phys (2014) 89:347–52. doi: 10.1016/j.ijrobp.2014.02.021 24721588

[16] Shields CL Manalac J Das C Ferguson K Shields JA . Choroidal melanoma: clinical features, classification, and top 10 pseudomelanomas. Curr Opin Ophthalmol (2014) 25 (3):177–85. doi: 10.1097/ICU.0000000000000041 24614143

[17] Duker JS Kaiser PK Binder S De Smet MD Gaudric A Reichel E . The international vitreomacular traction study group classification of vitreomacular adhesion, traction, and macular hole. Ophthalmology (2013) 120:2611–9. doi: 10.1016/j.ophtha.2013.07.042 24053995

[18] Evans JR Schwartz SD McHugh JDA Thamby-Rajah Y Hodgson SA Wormald RPL . Systemic risk factors for idiopathic macular holes: a case control study. Eye (1998) 12:256–9. doi: 10.1038/eye.1998.60 9683950

[19] Smiddy WE Flynn HW . Pathogenesis of macular holes and therapeutic implications. Am J Ophthalmol (2004) 137:525–37. doi: 10.1016/j.ajo.2003.12.011 15013877

[20] Bonnin N Cornut P Chaise F Labeille E Manificat HJ Feldman A . Spontaneous closure of macular holes secondary to posterior uveitis: case series and a literature review. J Ophthalmic Inflammation Infect (2013) 3:1–7. doi: 10.1186/1869-5760-3-34 PMC3605119 23514634

[21] Garoon RB Shields CL Kaliki S Shields JA . Cystoid macular edema as the initial manifestation of choroidal melanoma. Oman J Ophthalmol (2012) 5:187–9. doi: 10.4103/0974-620X.106104 PMC3574517 23440248

[22] Michael JG Venecia GDE . Retinal trypsin digest study of cystoid macular edema associated with peripheral choroidal melanoma. Am J Ophthalmol (1995) 119:152–6. doi: 10.1016/S0002-9394(14)73867-X 7832220

[23] Puusaari I Heikkonen J Summanen P Tarkkanen A Kivela T . Iodine brachytherapy as an alternative to enucleation for large uveal melanomas. Ophthalmology (2003) 6420:2223–34. doi: 10.1016/S0161-6420(03)00661-4 14597534

[24] Dunavoelgyi R Dieckmann K Gleiss A Sacu S Kircher K Georgopoulos M . Local tumor control, visual acuity, and survival after hypofractionated stereotactic photon radiotherapy of choroidal melanoma in 212 patients treated between 1997 and 2007. Int J Radiat Oncol Biol Phys (2011) 81:199–205. doi: 10.1016/j.ijrobp.2010.04.035 20675066

[25] Jouhi S Al-Jamal RT Täll M Eskelin S Kivelä TT . Presumed incipient choroidal melanoma: Proposed diagnostic criteria and management. Br J Ophthalmol (2021) 1–6. doi: 10.1136/bjophthalmol-2020-318658 34666992

[26] Puusaari I Heikkonen J Kivelä T . Ocular complications after iodine brachytherapy for large uveal melanomas. Ophthalmology (2004) 111:1768–77. doi: 10.1016/j.ophtha.2004.03.027 15350335

[27] Mashayekhi A Shields CL Lee SC Marr BP Shields JA . Retinal break and rhegmatogenous retinal detachment after transpupillary thermotherapy as primary or adjunct treatment of choroidal melanoma. Retina (2008) 28:274–81. doi: 10.1097/IAE.0b013e318145abe8 18301033

[28] Eibenberger K Dunavoelgyi R Gleiss A Sedova A Georg D Poetter R . Hypofractionated stereotactic photon radiotherapy of choroidal melanoma: 20-year experience. Acta Oncol (2021) 60:207–14. doi: 10.1080/0284186X.2020.1820572 32969745

[29] Bianciotto C Shields CL Pirondini C Mashayekhi A Furuta M Shields JA . Vitreous hemorrhage after plaque radiotherapy for uveal melanoma. Retina (2012) 32:1156–64. doi: 10.1097/IAE.0b013e3182340cc1 22366905

[30] Relimpio-López I Garrido-Hermosilla AM Espejo F Gessa-Sorroche M Coca L Domínguez B . Clinical outcomes after surgical resection combined with brachytherapy for uveal melanomas. J Clin Med (2022) 11:1616. doi: 10.3390/jcm11061616 35329942 PMC8956023

[31] Shabto JM Bergstrom CS Wells JR . Extraocular extension of a regressed choroidal melanoma after vitrectomy and scleral buckle for rhegmatogenous retinal detachment. Ophthalmology (2022) 129:275. doi: 10.1016/j.ophtha.2021.07.029 35190094

